# Supplementary material for: Pediatric Patients with Stage IV Rhabdomyosarcoma Significantly Benefit from Long-Term Maintenance Therapy: Results of the CWS-IV 2002 and the CWS DOK IV 2004-Trials
Source: Cancers (Basel). 2023 Mar 30;15(7):2050. doi: 10.3390/cancers15072050 (PMC10093505; doi:10.3390/cancers15072050)
Supplement: Supplementary file 1 [file cancers-15-02050-s001.zip › cancers-2279567-supplementary.pdf]

Supplementary Materials

Table S1. Patient characteristics by different final treatment approaches and risk factors. P values determined using Fisher's exact test, chi-square test or Kruskal-Wallis test as appropriate comparing the excluded, LTMT, HDCT and alloHSCT groups.

Patient characteristics by final treatment *					
	**Excluded**	**LTMT**	**HDCT**	**alloSCT**	**P**
N (%)	53 (30)	89 (51)	13 (7)	21 (12)	
**Sex, N (%)**					0.096
Female	21 (40)	49 (55)	6 (46)	6 (29)	
Male	32 (60)	40 (45)	7 (54)	15 (71)	
**Age, N (%)**					
Median (range), y	11 (0 – 21)	12 (2 – 20)	14 (2 – 18)	12 (4 – 20)	0.707
≤ 1 y	4 (8)	0	0	0	
1 – 9 y	22 (42)	36 (60)	4 (31)	7 (33)	
≥ 10	27 (51)	53 (40)	9 (69)	14 (67)	
**Histology, N (%)**					0.240
Embryonal RMS	20 (38)	30 (34)	4 (31)	4 (19)	
Alveolar RMS	23 (43)	43 (48)	8 (62)	16 (76)	
Other	10 (19)	16 (18)	1 (8)	1 (5)	
**Primary site of tumor, N (%)**					0.808
Favorable	6 (11)	7 (8)	1 (8)	1 (5)	
Unfavorable	47 (89)	82 (92)	12 (92)	20 (95)	
**Tumor size, N (%)**					0.471
≤ 5 cm	11 (21)	13 (15)	1 (8)	5 (24)	
> 5 cm	40 (75)	71 (80)	11 (85)	13 (62)	
Unknown	2 (4)	5 (6)	1 (8)	3 (14)	
**Number of metastases, N (%)**					0.145
≤ 2	39 (74)	64 (72)	6 (46)	12 (57)	
≥ 3	14 (26)	25 (28)	7 (54)	9 (43)	
**Bone or bone marrow involvement, N (%)**					<0.001
Yes	24 (45)	33 (37)	9 (69)	18 (86)	
No	29 (55)	56 (63)	4 (31)	3 (14)	
**Oberlin score, (%)**					0.019
≤ 1	14 (26)	29 (33)	1 (8)	1 (5)	
≥ 2	39 (74)	60 (67)	12 (92)	20 (95)	
**Period of start initial chemotherapy**					0.643
Median	Mar 2006	Oct 2006	Jul 2005	Aug 2006	
Range	Feb 2003 - May 2010	Nov 2002 - Jul 2010	Aug 2003 - May 2009	Apr 2003 - Feb 2009	
**Protocol, N (%)**					0.562
CWS-IV-2002	8 (15)	15 (17)	2 (15)	6 (29)	
CWS DOK IV 2004	45 (85)	74 (83)	11 (85)	15 (71)	
**Initial chemotherapy, N (%)**					0.195
Window TC/VAIA	33 (62)	48 (54)	6 (46)	14 (67)	
CEVAIE	16 (30)	40 (45)	6 (46)	7 (33)	
Others	4 (8)	1 (1)	1 (8)	0	

**Time between start of initial chemotherapy and final treatment, days**					0.001
Median	-	258	226	228	
Range	-	133 - 472	167 - 320	170 - 344	
**Period of start final treatment**					0.537
Median	-	Jul 2007	May 2006	May 2007	
Range	-	Jun 2003-May 2011	Mar 2004 - Jan 2010	Dec 2003 - Sep 2009	

* The patient characteristics of excluded patients (n=53) were similar to total patients

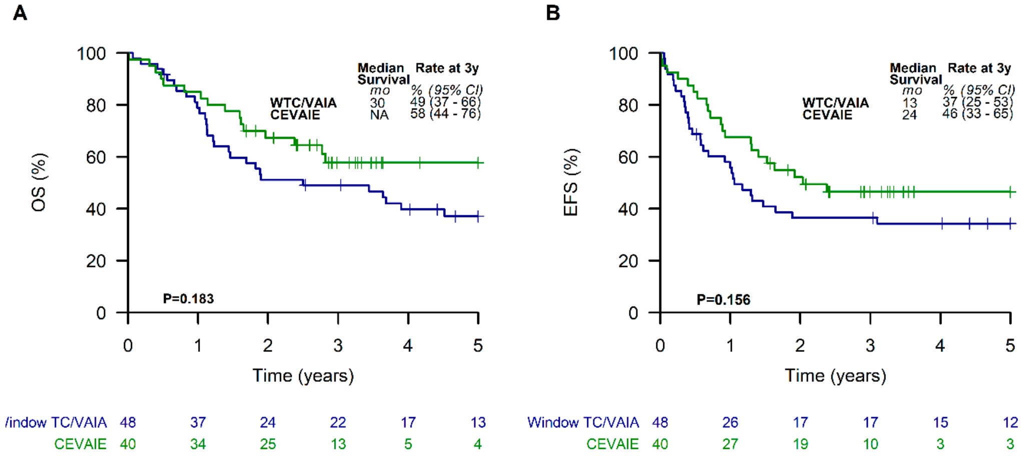

Figure S1. Overall survival (**A**) and event-free survival (**B**) of patients receiving a metronomic long-term maintenance therapy with respect to the different conventional chemotherapy regimens.

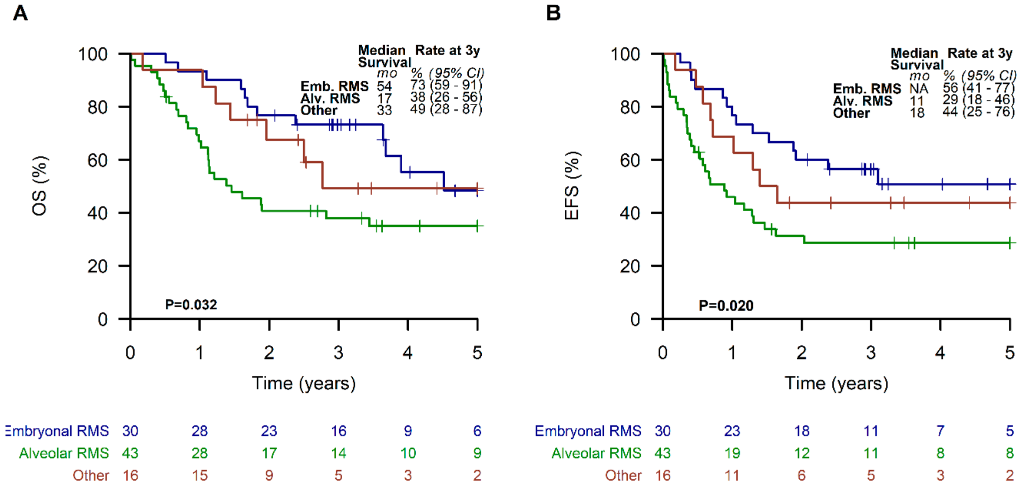

Figure S2. Overall survival (**A**) and event-free survival (**B**) of all patients with regard to the different histological subtypes.
